# A new species of *Erythrostemon* (Leguminosae, Caesalpinioideae) from the western Río Balsas Depression, Mexico

**DOI:** 10.3897/phytokeys.76.10921

**Published:** 2017-01-09

**Authors:** Solange Sotuyo, José Luis Contreras-Jiménez, Gwilym P. Lewis

**Affiliations:** 1 Departamento de Botánica, Instituto de Biología, Universidad Nacional Autónoma de México. Circuito Exterior s/n, Ciudad Universitaria, Copilco, Coyoacán. A.P. 70-367 México, Distrito Federal. C.P. 04510; 2 Facultad de Arquitectura, Benemérita Universidad Autónoma de Puebla. 4 Sur 104. Col. Centro. CP 72000. Puebla, Puebla; 3 Comparative Plant and Fungal Biology Department, Royal Botanic Gardens, Kew, Richmond, Surrey, TW9 3AB, U.K.

**Keywords:** Caesalpinia group, Leguminosae, Erythrostemon, Fabaceae, Neotropics

## Abstract

A new legume species from a seasonally dry forest of the Western Río Balsas Depression, in the states of Guerrero and Michoacán, Mexico, *Erythrostemon guevarafeferii*, is herein described and illustrated. The new species shows morphological affinities with *Erythrostemon hintonii*, from which it is distinguished in having fewer leaflets per pinna, mature leaflets disposed toward the upper half of the pinnae rachises, long inflorescences on curved slender peduncles, abundant red glands on its flowers and inflorescences, and its fruit glabrous with red stipitate glands at maturity. A taxonomic key to the Río Balsas Depression species of *Erythrostemon* is included.

## Abstract (es)

Una nueva especie de leguminosa de la Depresión Occidental del Río Balsas en los estados de Guerrero y Michoacán, *Erythrostemon guevarafeferii*, se describe e ilustra. La nueva especie muestra afinidades morfológicas con *Erythrostemon hintonii*, de la que se distingue por tener menos pinnas, folíolos maduros glabros dispuestos hacia la mitad superior de las pinnas, las inflorescencias largas sobre pedúnculos delgados que se curvan y flores con abundantes glándulas; legumbre glabra o con glándulas estipitadas rojas cuando esta madura. Se incluye una clave taxonómica de las especies de *Erythrostemon* que habitan en la Depresión del Río Balsas.

## Introduction

*Erythrostemon* was re-circumscribed by Gagnon et al. (2016). The neotropical genus currently includes a total of 31 species of woody shrubs or small to medium-sized trees. Species distributions follow a bicentric amphitropical distribution pattern in México, Central America and the Caribbean, Brazil, Argentina, Bolivia, Chile and Paraguay (Lewis 1998; Gagnon et al. 2013; Gagnon et al. 2016). They grow in a wide range of habitats including seasonally dry tropical forests, caatinga vegetation, deserts, yungas-puna transition zones, and chaco-transition forests.

Species of *Erythrostemon* are distinctive by various combinations of several morphological traits (a reference for this cited here). Leaflets typically are either eglandular or have conspicuous black sessile glands along their margin that renders the margins slightly crenulated. Flowers have sepals that are ovate-lanceolate and their petals bending and either yellow, red, pink or orange, the corolla sometimes laterally compressed. The androecium and gynoecium are free from the calyx and the ovary is eglandular or covered in gland-tipped trichomes. Legumes are typically oblong-elliptic pods with papery to slightly woody valves that are chartaceous or slightly woody, glabrous to pubescent, eglandular or with stipitate glands.

During a revision of *Caesalpinia* sensu lato in Guerrero and Michoacán, several specimens identified in herbaria as *Caesalpinia hintonii* Sandwith (a species recently transferred to the genus *Erythrostemon*, Gagnon et al. 2016) proved to be morphologically distinct. Taking into account the above morphological diagnostic characters of *Erythrostemon*, a detailed study indicated that these specimens belong to that genus, although they do not match the description of any previously described species. The overall morphology of the new taxon most closely resembles *Erythrostemon hintonii* (Sandwith) E. Gagnon & G.P. Lewis, with which it shares a similar distribution area in the Río Balsas Depression. The new species is herein described as *Erythrostemon guevarafeferii*.

## Taxonomy

### Erythrostemon guevarafeferii

Taxon classificationPlantaeFabalesFabaceae

J.L.Contreras, S.Sotuyo & G.P.Lewis sp. nov.

urn:lsid:ipni.org:names:77159617-1

Figure 1

#### Type.

México, Michoacán. Cerca de la cortina de la presa El Infiernillo *J.L. Contreras 3111* (Holotype: MEXU; Isotypes: FCME, K, MEXU).

*Erythrostemon hintonii* affinis sed glabra, structuris floralibus omnino glanduliferis, foliis minoribus cum minus foliolis fasciculatus in dimidio superiore rhachidis pinnae, floribus brevioribus, sepalis laete ochraceis et petalis salmoneis, pedicellis articulatis in dimidio superiore longitudinis, legumine omnino glabro et stipitatis glandulis rubris obducto. Etiam differt inflorescentias arcuatas et pendulas cum pedicellis florum gracilibus, fere horizontalibus vel reflexis, tortis itaque floribus resupinatis.

Similar to *Erythrostemon hintonii*, but glabrous, all flower structures glandular, leaves smaller with fewer leaflets, flowers shorter, sepals light yellow-ochre and petals salmon coloured, pedicels articulated at, or above, their middle, legume completely glabrous and with stipitate red glands. Also differing in the curved and/or pendulous inflorescences with the flowers on slender, horizontal or reflexed, twisted pedicels that render them resupinate.

#### Description.

Small tree or shrub, 2–6 m tall, bark grey pruinose, exfoliating, young branches reddish grey pruinose. Leaves bipinnate, (7.5–) 10–20 (–22) cm long; stipules triangular-acuminate, caducous, 2–3 × 1.2–1.5 mm, margin with red stipitate glands, white hairy on both surfaces; petiole (2–) 3.0–6.5 (–7.3) cm long, pubescent or glabrescent when mature; rachis (3–) 5.0–14.5 (–15.7) cm long, indumentum similar to that of the petiole; pinnae (5–) 7–13 per leaf, (1.6–) 2–6 (–7.7) cm long, densely pubescent when young, glabrescent when mature; leaflets (3) 4–7 (–10) jugate, clustered on the upper half of the pinnae rachises, obovate, elliptic or oblong-elliptic (6–) 10–20 (–25) × (4–) 5–11 (–13) mm, base rounded, slightly oblique, margin entire and lacking glands, or with sunken black punctate glands that render the margin slightly crenulate, apex obtuse to rounded, pubescent or glabrescent at maturity. Inflorescence an erect or pendulous (the peduncles curved downwards), terminal or axillary raceme or panicle (with few branches near the base), (6–) 10–36 cm long, densely white pubescent, and with red glands; bracts ovate-caudate, caducous, (2.5–) 3.2–5.0 (–5.7) × (1–) 1.3–2.0 (–2.3) mm, margin with or without glands, densely white pubescent on both surfaces; pedicels slender, erect-patent, (6–) 7.5–11.0 (–16.5) mm long, articulated at or above the middle, densely white pubescent, sometimes twisted, so that the flowers are resupinate (although then presented with the median petal uppermost on the pendulous inflorescences). Calyx light yellow-ochre, densely pubescent, tube obliquely obconic, laterally compressed, (4.3–) 4.7–6.0 × (2.1–) 2.5–3.5 (–4) mm; adaxial sepals ovate to oblong-ovate, 5.5–7.0 × 2.5–4.1 mm, slightly concave, the lateral sepals oblong-ovate, 5.4–7.0 mm × 2.7–4.0 mm; abaxial sepal cymbiform, (5.7–) 6–7.2 (–7.4) × (3.6–) 3.8–5.0 mm, all sepals with an entire margin, glandular ciliate and with an acute apex, puberulent on adaxial surface; petals salmon-coloured, clawed; adaxial petal ovate, 5.2–7.5 × 3.5–5.5 mm, base cordate, margin entire, apex obtuse, rounded, with stipitate glands near the claw apex on the abaxial surface, pilose on the adaxial surface; claw 1.8–3.0 mm long, sigmoid curved, ciliate, pilose and glandular-stipitate on abaxial surface or glabrous and sparsely stipitate-glandular; lateral petals ovate, 5.6–7.5 (–8) × 4.2–6 (–6.5) mm, base obtuse, margin glandular ciliate up to ⅕ of its length, apex rounded or obtuse, stipitate-glandular on abaxial surface, the abaxial petals ovate (6–) 6.5–8.5 × 3.7–5.5 (–5.7) mm, base oblique, margin entire, glandular ciliate from the base up to ⅕ of their length, apex obtuse, the claw ciliate and pilose on its abaxial surface; stamens curved, filaments (6.5–) 7.5–9.5 (–10) mm long, flattened at the base, densely villous to ¾ of their length, the upper third with lime green stipitate glands; anthers oblong-elliptic, (1.2–) 1.3–1.8 × (0.8–) 0.9–1.2 (–1.3) mm, erect at anthesis; ovary (1.7–) 2–3 (–3.5) mm long borne on a stipe 0.5 mm long, densely sericeous and with green cupuliform glands (or these absent); style curved, of different lengths in individual flowers, either short, 2.5–3.5 mm long, or well developed and 4.0–6.5 mm long, pilose for ½ of its length from base; stigma porate laterally; ovules 2 (–3) per ovary. Legume falcate, (3.7–) 4.3–5.7 × 1.1–1.7 cm, chartaceous, brown-yellow to vinaceous, densely to sparsely pubescent or glabrous when mature, with red stipitate glands or these glands absent, sutures densely pubescent, elastically dehiscent; seeds 1–2 (–3) per fruit, obovate, (8.6–) 9.5–10.5 (–11) × 7.5–9.6 × (1.7–) 1.9–2.2 (–2.4) mm, olive-brown, nitid.

**Figure 1.**
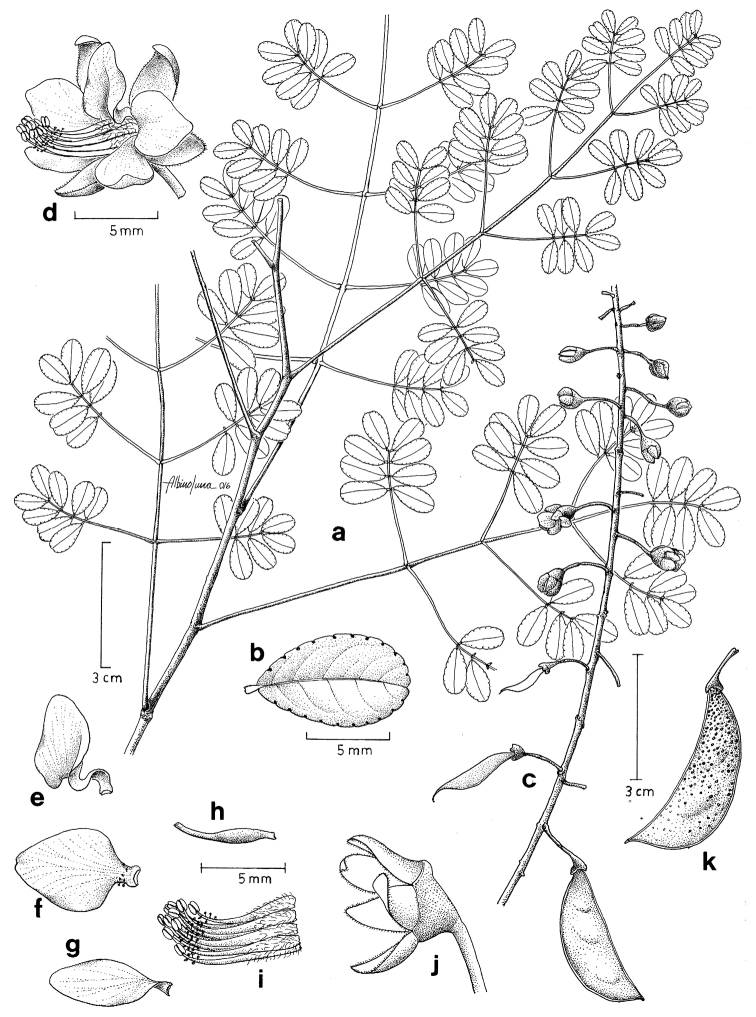
*Erythrostemon guevarafeferii*, **a** bipinnate leaves **b** leaflet upper surface showing marginal glands **c** inflorescence with immature fruits developing at base **d** flower **e** adaxial (median) petal **f** lateral petal **g** abaxial petal **h** gynoecium **i** stamens **j** calyx **k** fruit showing the red stipitate glands as black dots. (**a**–**j** drawn from *Contreras-Jiménez et al.* 3111, holotype MEXU **k** from *Contreras-Jiménez* 2860). Drawn by Albino Luna.

#### Habitat.

Seasonally dry tropical forest on rocky slopes, locally common in secondary vegetation along roads, on alluvial soils near seasonal or permanent streams.

#### Distribution and phenology.

Known only from the western region of the Río Balsas Depression, in the states of Guerrero and Michoacán. Flowering from February to April and in fruit from February to May.

Selected specimens examined. MÉXICO. **Michoacán**: Municipio de Arteaga: El Infiernillo, cerca de la cortina de la presa: *Nuñez & Silva 3905* (MEXU); *J.C. Soto 1331 & S. Zárate* (MEXU); *J.C. Soto 3694* (MEXU); *Sotuyo et al. 41,46,47,48* (K, MEXU); 23 km por el camino a Infiernillo a partir de la carretera Apatzingan-Lázaro Cárdenas: *J.L. Contreras 2060, 2838* (MEXU). Municipio de Nocupétaro: San Antonio de los Muertos; *J.C. Soto 3905 & G. Silva* (MEXU). **Guerrero**: Municipio de la Unión: 5 km al S de Colmeneros, camino a Coahuayutla: *J.L. Contreras 2371, 2372* (FCME); Cerro Prieto, 12 km al E de la Garita: *J.L. Contreras 2388* (FCME); Zihuatanejo, 85 km aprox. NW, on road 22 km to N of La Unión and 7 km to N of Las Juntas del Río towards Santa María: *D.J. Macqueen & A. Nileshwar 446* (K).

#### IUCN Red List category.

We recommend that *Erythrostemon guevarafeferii* be given a conservation assessment of Vulnerable [VU (B1b-iii)], in accordance with IUCN (2012) categories and criteria. The extent of occurrence (EOO) of *Erythrostemon guevarafeferii* is estimated to be over 2424.18 km^2^, well below the 20,000 km^2^ upper limit for Vulnerable status under criterion B1, but its area of occupancy (AOO) is estimated to be less than 10 km^2^ (the limit for Endangered status under criterion B2). The species is currently known from three discontinuous populations in the states of Michoacán and Guerrero, these separated by differing habitat type, human settlement and agricultural land. *Erythrostemon guevarafeferii* is known in only one protected area, the Reserva de la Biosfera Zicuirán-Infiernillo in Michoacán. The preferred habitat of the species is potentially threatened by future settlement and agricultural activities, as well as by environmental problems associated with drug trafficking organizations.

#### Etymology.

The species epithet is dedicated to Fernando Guevara Fefer who recently passed away. Friend, botany teacher and researcher at the Universidad Michoacana de San Nicolás de Hidalgo, he was interested in the genus *Bursera* and in floristic and vegetation studies within Michoacán, particularly in the Infiernillo region where the type specimen was collected.

## Discussion

*Erythrostemon guevarafeferii* differs from *Erythrostemon hintonii* in being glabrous at maturity and by sometimes having glands on floral structures and fruits. It has smaller leaves with (5–) 7–13 pinnae per leaf and leaflets (3–) 4–7 (–10) jugate, these on the upper half of the pinna rachis. Flowers are smaller (1.2–1.6 cm long, including the calyx) with light yellow-ochre sepals and salmon-pink petals, its pedicels are articulated at or above their middle. Its legume is glabrous when mature and sometimes has red stipitate glands.

*Erythrostemon hintonii* differs by having leaves with 3–9 pinnae per leaf and leaflets 4–6 jugate, these distributed along the length of the pinna rachis, flowers 2 cm long (including the calyx) with red-purple sepals and purple-red petals, pedicels that are articulated near the base of the calyx tube, and a legume that is pubescent and has dark red-brown sessile glands. Some individual plants of *Erythrostemon guevarafeferii* have glandular fruits while others in the same locality do not. The species is characterized by its arched and pendulous inflorescences, with slender, nearly horizontal pedicels (or these reflexed and sometimes twisted, so that the flowers are resupinate). Resupinate flowers on a pendulous inflorescence renders them, once again, in their usual position with the median (standard) petal uppermost.

Field and laboratory observations reveal two kinds of flower, with either a short or long pistil, as also observed in *Erythrostemon epifanioi* (J.L. Contr.) E. Gagnon & G.P. Lewis (as *Caesalpinia epifanioi* J.L. Contr.; Lewis, 1998) and in another legume genus, *Tylosema esculentum* (Burch.) A. Schreib., which displays functional heterostyly (Hartley et al. 2002). Flowers with short pistils are functionally male and flowers with long pistils are hermaphrodite. We hypothesise that the occurrence of both flower forms in the same inflorescence ensures that not too many flowers on each inflorescence set fruit (which would put a strain on maternal resources, as well as mechanically over-loading the peduncle) and that pollinators continue to move pollen throughout or between populations of the species, thus promoting cross-pollination. Andromonoecy, with a labile sex change of flowers from hermaphrodite to functionally male (the gynoecium development is suppressed in flowers higher up an inflorescence only if the lower flowers are successfully pollinated and fruits are set) has been observed in *Erythrostemon calycina* (Benth.) L.P. Queiroz (as *Caesalpinia calycina* Benth.) in Brazil (Gibbs et al. 1999).

### Taxonomic key to the species of *Erythrostemon* in Rio Balsas Depression

**Table:** 

1	Shrubs or herbaceous plants; leaflets without black glands along margin; flowers deflexed; glandular stamen filaments curved, twice the length of the petals; legume 4.7–7.1 cm long; Sierra Madre del Sur, Guerrero and Oaxaca, 1400–2000 m elevation	***Erythrostemon laxus***
–	Trees; leaflets with black glands along margin; flowers reflexed; stamen filaments falcate or curved, equalling the length of the petals; legume 3–6 cm long; seasonally dry forest of the Río Balsas Depression, 100–1200 m elevation	**2**
2	Leaves with 3–5 pairs per pinnae, leaflets 2–6 pairs per pinna; inflorescence a raceme, on a short woody brachyblast	**3**
–	Leaves with 7–13 pairs per pinnae, leaflets 4–11 pairs per pinna; inflorescence a terminal or axillary raceme or panicle, peduncles branched near base	**4**
3	Leaflets 2–3 pairs per pinna; pedicels 6.5–12.5 mm long, pubescent; eastern region of Río Balsas Depression (Ozomatlán, Guerrero)	***Erythrostemon epifanioi***
–	Leaflets 4–6 pairs per pinna; pedicels c. 7 mm long, densely stipitate-glandular; Valle de Tehuacán-Cuicatlán (Puebla and Oaxaca)	***Erythrostemon melanadenius***
4	Pedicels articulated below the middle; petals yellow; leaflets oblong-elliptic	***Erythrostemon macvaughii***
–	Pedicels articulated at or above the middle; petals pink, red or scarlet; leaflets ovate or oblong	**5**
5	Fruit with red cupuliform glands or these absent; standard petal red or salmon coloured, stamen filaments pubescent on basal ½ to ¾, sometimes stipitate-glandular; western part of Río Balsas Depression (Guerrero and Michoacán)	**6**
–	Fruit with green pixie-cup (or doughnut-shaped) glands; standard petal pale pink with a red blotch at the lamina base on the inner surface; stamen filaments with green glands; western Río Balsas Depression (Guerrero, Oaxaca and Puebla)	***Erythrostemon oyamae***
6	Leaflets obovate to elliptic, inserted along the pinna rachis; bracts erect or incurved; stamen filaments 13–15 mm long; legume with red cupuliform glands; seasonally dry forest in Guerrero and Michoacán	***Erythrostemon hintonii***
–	Leaflets oblong-elliptic, inserted on the upper half of the pinna rachis; bracts ovate-caudate; stamen filaments 6.5–10 mm long; legume with or without red cupuliform glands; Infiernillo region in the Río Balsas Depression (Guerrero and Michoacán)	***Erythrostemon guevarafeferii***

## Supplementary Material

XML Treatment for Erythrostemon guevarafeferii
